# Arsenic Speciation and Distribution in Three *Hebeloma* Species: Insights into Arsenic Handling and Transformation in Mycorrhizal Fungi

**DOI:** 10.1007/s00248-026-02780-9

**Published:** 2026-05-06

**Authors:** Jan Šnábl, Antonín Kaňa, Gabriela Pelešková, Jana Steinbauerová, Petr Rudolf, Jan Borovička, Tereza Leonhardt, Jan Sácký

**Affiliations:** 1 Department of Biochemistry and Microbiology, Institute of Chemical Technology, Technická 3, Prague, Prague 6, 166 28 Czech Republic; 2 Nuclear Physics Institute, Czech Academy of Sciences, Řež 292, Husinec, 25068 Czech Republic; 3 Institute of Geology, Czech Academy of Sciences, Rozvojová 269, Prague 6, 16500 Czech Republic; 4 Department of Analytical Chemistry, University of Chemistry and Technology, Technická 5, Prague, Prague, 166 28 Czech Republic

**Keywords:** Organoarsenicals, Arsenobetaine, Methylation, Arsenic biotransformation, Ectomycorrhizal fungi

## Abstract

Arsenic accumulation and biotransformation in fungi remain poorly understood, particularly in mushrooms. This study investigated arsenic distribution, speciation, and detoxification mechanisms in three mushroom species of the genus *Hebeloma*: *H. bulbiferum*, *H. sinapizans*, and *H. mesophaeum*. Wild collected fruit bodies and laboratory mycelial cultures were analysed using size-exclusion chromatography (SEC) and ion-pair reversed-phase ICP-MS to determine total arsenic concentrations and speciation patterns. *H. bulbiferum* exhibited the highest arsenic accumulation in fruit bodies (up to 563 mg kg⁻¹ dry mass), predominantly as dimethylarsenate, whereas *H. sinapizans* accumulated less total As (up to 41.2 mg kg⁻¹ dry mass), comprising mainly arsenobetaine and dimethylarsenate, and *H. mesophaeum* (up to 4.2 mg kg⁻¹ dry mass) was dominated by inorganic arsenic and arsenocholine. SEC revealed that arsenic was present as low-molecular-weight fractions, although a minor protein-associated peak was observed in *H. sinapizans*, suggesting a possible presence of an arsenic-binding protein. Mycelial cultures demonstrated species-specific tolerance to arsenate and the ability to transform inorganic arsenic into organic arsenicals, with varying degrees of arsenate reduction, arsenite efflux, and methylation. In particular, *H. sinapizans* and *H. mesophaeum* exhibited a higher degree of As(III) efflux than *H. bulbiferum*, indicating a more efficient As reduction and export. Notably, some organoarsenicals, dimethylarsenate and trimethylarsine oxide, were actively excreted into the growth medium, indicating a role for mushroom mycelia in environmental arsenic cycling. These findings highlight distinct arsenic detoxification strategies in *Hebeloma* species, reveal fungal *de* *novo* arsenobetaine synthesis, and provide insights into arsenic transformation and sequestration in ectomycorrhizal mushrooms.

**Supplementary Information:**

The online version contains supplementary material available at 10.1007/s00248-026-02780-9.

## Introduction

Arsenic (As) is a trace element (metalloid) that has well-documented toxic effects on living organisms, including humans, where chronic exposure is linked to diverse pathologies, including multi-organ failure, dermatological lesions, and various forms of cancer, making it a premier global concern for public health and environmental safety [1, 2]. Thus, prokaryotes and eukaryotes, including fungi, evolved biochemical mechanisms to manage As toxicity [3]. Arsenic enters the environment through natural processes, such as volcanic activity and rock weathering, as well as through anthropogenic activities, including mining, smelting, and coal combustion. In the terrestrial environment, As exists in various chemical forms, with inorganic species predominating [4] and generally being more toxic to living organisms than organic species [5]. Arsenic toxicity is primarily driven by its two biologically relevant oxidation states, As(V) and As(III). As(V) acts as a phosphate analog that disrupts essential phosphorylation processes, while As(III) binds to critical thiols to inactivate key enzymes [3].

Common organoarsenicals include methylarsenate [MA(V)] and methylarsenite [MA(III)], dimethylarsenate [DMA(V)] and dimethylarsenite [DMA(III)], trimethylarsine [TMA(III)], trimethylarsine oxide (TMAO), arsenocholine (AC), arsenobetaine (AB), and the tetramethyl arsonium ion (TETRA). MA(III) and DMA(III) are highly toxic to humans, though they are less stable than other As species. Yet they remain relatively stable in the reducing cellular environment. However, these trivalent As species may be partially oxidized to MA(V) and DMA(V) during sample collection, storage, and processing. Therefore, trivalent As species are rarely detected in environmental samples [5].

Organoarsenicals are presumably the products of biotransformations that serve as detoxification mechanisms in the biota. Many organoarsenicals, especially the pentavalent forms, are less toxic than their trivalent counterparts [5]. This detoxification process, originally described by Challenger [6], involves the conversion of inorganic arsenic (iAs) into organoarsenicals and uses redox enzymes, methyltransferases, and other biosynthetic pathways to generate less toxic or non-toxic forms, such as arsenosugars and arsenolipids. This process is likely present in all living organisms, from bacteria to humans. Mushrooms (also called macrofungi or macromycetes) are known to naturally accumulate As in their fruit bodies, sometimes at concentrations exceeding 1,000 mg kg^− 1^ in dry weight (d.w.) [7, 8]. The most common organoarsenicals found in naturally collected mushrooms are MAs, DMAs, AB, AC, and TMAO [9]. However, several previously unknown organic As compounds, such as arsenocholine-*O*-sulfate, arsenobetaine amide, trimethyl(2-carboxyethyl)arsonium, and trimethyl(3-hydroxypropyl)arsonium have recently been identified in fungal stromata and fruit bodies [10–12]. These findings raised the question of whether, like bacteria and microscopic fungi, mushrooms can transform iAs into organic species [13] or absorb organoarsenicals from the environment. It was speculated that the organoarsenicals found in mushrooms, especially AB and TMAO, may also be biosynthesized by symbiotic microorganisms and taken up by the mushroom during sporocarp formation [14, 15].

Early *in vitro* experiments on As in mushrooms [16] revealed that the mycelium of *Agaricus moelleri* (reported as *A. placomyces* in the study) could convert MA(V) to DMA(V) when grown on a sterilized agar substrate containing various As species. However, no other conversions were observed. This finding was unexpected because AB was identified as the primary species in naturally occurring fruit bodies of *A. moelleri* [17]. AB is the predominant As species in marine organisms, such as crabs, shrimps, and lobsters. Although As concentrations in seawater are very low (1–5 µg L^− 1^), marine animals contain much higher levels (in the mg kg^− 1^ range), with AB accounting for 75–100% of their total As content [18]. AB occurs in smaller and more variable amounts in freshwater organisms and lichens [19, 20] and has also been detected as a major As compound in mushrooms [9, 21]. The origin and formation mechanism of AB in the biota remain unclear; however, some research suggests that it arises from AC via microbial activity [22].

Soeroes [14] examined *Agaricus bisporus* grown on sterile wheat straw mixed with horse dung. The study revealed that AB and MA(V) were present in the fruit bodies, but not in the substrate. In contrast, iAs and DMA(V) were found in both the fruit bodies, where they comprised 98% of total As, and the substrate. Since the substrate was contaminated with As species even before inoculation with *A. bisporus* mycelium, it could not be definitively concluded whether DMA(V) is formed *de novo* by the fungus or is only absorbed from the substrate. *In vitro* studies on the mycelia of *A. bisporus*, *Suillus luteus*, and *Sparassis crispa* have also not provided convincing evidence of iAs transformation in mushrooms [15, 23]. Only the *S. crispa* strain showed partial conversion of As(V) to TMAO, which was, however, an unexpected result given that wild-growing fruit bodies of *S. crispa* are known to contain mainly AC [12, 24].

Since the ability of macrofungi to independently synthesize organoarsenicals remains a subject of ongoing debate, this study evaluates the intrinsic capacity of mushroom mycelia to transform iAs. By employing an axenic laboratory system with As(V) as the sole As source, we provide definitive evidence that mycelia of three ectomycorrhizal *Hebeloma* species independently biotransform iAs into complex organic species, including AB, DMA(V), and TMAO. The presence of these same compounds in wild-growing fruit bodies confirms the ecological relevance of our laboratory observations. Furthermore, our results demonstrate that these mycelia actively release organoarsenicals into the surrounding substrate, revealing a previously unconfirmed role for macrofungi in terrestrial As cycling.

## Materials and Methods

### Mushrooms and Mycelia Collection

Fruit bodies of *Hebeloma bulbiferum* and *Hebeloma sinapizans* were collected in *Carpinus betulus* plantation on Silurian limestone bedrock at Prague-Velká Chuchle, Czechia, from a locality of circa 20 × 20 m. Fruiting bodies of *Hebeloma mesophaeum* correspond to the sample B-269 used in an earlier study [25].

The dikaryotic mycelial isolates were prepared from the axenic explants from a freshly broken fruit body flesh of all investigated species. Briefly, the specimens were cleaned of debris and dissected to excise internal pileus tissue, avoiding contact with external fungal surfaces. Sterile forceps were used to transfer a small portion (~0.1–0.5 cm^3^) of pileus internal tissue onto potato dextrose (PD) agar plates with 100 mg L^− 1^ ampicillin to prevent bacterial contamination. The plates were incubated at 25 °C in the dark until fungal growth occurred. Freshly grown mycelia were subcultured onto PD agar plates for further storage and experiments. The ITS rDNA sequences of the mycelial isolates have been deposited in GenBank under the accession numbers PV902627.1, PV747898.1, and HF678210.1. The portions of the fruit body biomass to be used for the As speciation analysis and/or molecular work were stored fresh at -80 °C or fixed by lyophilization and stored at -80 °C. Several collections of both species from the exact sampling site have been documented in the fungarium of the Mycological Department of the National Museum in Prague under the accession numbers PRM 923858, PRM 861164, and PRM 954676 (*H. bulbiferum*), PRM 860840 and PRM 954677 (*H. sinapizans*), and PRM 899241 (*H. mesophaeum*). To document the As accumulation ability of the selected *Hebeloma* species, several collections from this site were harvested over the period of 2010 to 2020. The fruit bodies were cleaned from substrate debris, transported to the laboratory in paper bags, rinsed with distilled water, dried, milled, and analyzed for As content by instrumental neutron activation analysis (INAA) under the conditions described in Borovička [26]. To document the environmental conditions at the sampling site, three soil samples (0–10 cm depth, excluding raw organic debris) were collected, processed, and analyzed for total As content by INAA as described in Braeuer [27] (INAA results are available in Supplementary Tables 1 and 2).

### Size Exclusion Chromatography of Fungal Extracts

Fruit body extracts and subsequent size-exclusion chromatography (SEC) were prepared and performed as described earlier [28]. Briefly, extract fractionation was performed using a Superdex Peptide 10/300 GL column (GE Healthcare). The mobile phase consisted of 50 mM HEPES and 25 mM KNO₃ (pH 7.3), delivered at a flow rate of 0.5 mL min^− 1^ by a Series 200 high-pressure pump (PerkinElmer, Shelton, USA). The As signal was monitored as the AsNH_2_^+^ ion to suppress ArCl^+^ interference using an inductively coupled plasma mass spectrometer (ICP-MS) NexION 5000 (PerkinElmer, Shelton, CT, USA).

### Arsenic Tolerance Assay

The As tolerance experiment on mycelium was conducted as described in Sácký et al. [29], using Na_2_HAsO_4_ · 7H_2_O as the As source, with selected concentrations of 0, 100, 250, 500, and 1000 µM. Briefly, mycelia were inoculated as ~ 3 mm³ blocks onto cellophane-covered agar plates and cultivated for 30 d. The resulting biomass was scraped, lyophilized, and weighed. The experiment was performed in triplicate, and mean dry weights were used to determine growth inhibition, expressed as the percentage reduction in biomass relative to the As-free control.

### Total as Content and as Speciation in Mycelia and Sporocarps

The As speciation and uptake assays were performed on sterile PD agar plates supplemented with 1 µM, 10 µM, and 100 µM Na_2_HAsO_4_ · 7H_2_O, or no As as a control. A ~ 3 mm^3^ agar block of *Hebeloma* mycelium from a 30-day-old culture was aseptically transferred to the center of a PD agar plate covered with a sterile cellophane membrane and incubated at 25 °C for 21 d. Arsenic concentrations and species were measured both in the mycelia and the PD agar medium, which was located under the mycelium, but separated from the mycelium by the cellophane membrane.

The total As content in the mycelia and fruit bodies was determined using ICP-MS. Samples were subjected to microwave-assisted digestion (SpeedWave 4, Berghof, Germany) in a Teflon^®^ vessel with 3 mL of concentrated nitric acid (Analpure grade, Analytika, Czech Republic). The temperature was ramped to 240 °C within 8 min and held for 5 min. The digest was then quantitatively transferred into a 50 mL volumetric flask. The analysis of the As species was performed according to the procedure described by Kaňa [30]. The species were separated by ion-pair reversed-phase chromatography (IP-RP) on an RP-C8 column (Purospher^®^ STAR C8, 250 × 4.6 mm, 5 μm; Merck, Darmstadt, Germany) using a mobile phase consisting of 1.0 g L^− 1^ sodium butane-1-sulfonate, 0.42 g L^− 1^ malonic acid, 0.22 g L^− 1^ TMAH, and 0.05% (v/v) methanol at a flow rate of 1 mL min^− 1^. The As signal was monitored as the AsNH_2_^+^ ion by ICP-MS. The amount of As in each mushroom species was determined based on the chromatographic peak areas. Quantification was performed using external calibration with standard solutions of the individual As species. Calibration curves were constructed by plotting peak area versus concentration of standard, and the concentrations of the respective species in the samples were calculated accordingly. Data were analyzed via one-way ANOVA and Tukey’s post hoc testing (*p* < 0.05) using the R functions aov() and TukeyHSD().

## Results and Discussion

### Arsenic Distribution and Speciation in *Hebeloma* Fruit Bodies

Based on molecular and morphological evidence, *H. bulbiferum* and *H. sinapizans*, both collected from the same locality, are placed in the section *Sinapizantia*, whereas *H. mesophaeum*, whose fruit bodies originated from a different but equally undisturbed site, belongs to the section *Hebeloma*, indicating a more distant evolutionary relationship and illustrating the broader phylogenetic divergence within *Hebeloma* [31, 32]. The bulk As concentration in the analyzed soil (21.6 ± 1.4 mg kg⁻¹ dry mass) corresponded to background values [1]. The three *Hebeloma* species exhibited strikingly different capacities to accumulate and transform As (Table 1; Supplementary Tables 1 and 2). Sporocarps of *H. bulbiferum* (194–563 mg kg⁻¹ dry mass) accumulated up to 20 times more As than *H. sinapizans* (5.4–41.2 mg kg⁻¹ dry mass) and *H. mesophaeum* (below 4.2 mg kg⁻^1^ dry mass). According to this data, *H. bulbiferum* acts as an As accumulator.

**Table 1:** Contents of identified and unknown (UNK) As species in fruit bodies of *Hebeloma* species (dry weight). n.d. = not detected

sample	Hebeloma species		As species (mg As kg^− 1^)									sum of As in species (mg kg^− 1^)	total As in extract (mg kg^− 1^)	total As in biomass (mg kg^− 1^)	extraction efficiency (%)
		DMA(V)		AB	TETRA	TMAO	MA(V)	AC	As(III)	As(V)	∑ UNK				
B-1527	H. bulbiferum*	416		n.d.	1.12	n.d.	0.21	0.15	n.d.	0.02	1.50	419	551	563	98
B-628	*H. bulbiferum*	138		n.d.	0.63	n.d.	0.18	n.d.	n.d.	0.03	0.35	139	169	186	91
B-1526	H. sinapizans*	0.63		4.24	n.d.	0.59	0.04	0.08	n.d.	0.04	0.37	6.00	8.91	11.7	76
PAKO-101	*H. sinapizans*	2.06		1.82	n.d.	0.19	0.12	0.18	n.d.	0.01	0.19	4.57	7.97	12.2	65
B-269	H. mesophaeum*	0.95		n.d.	n.d.	n.d.	n.d.	1.13	1.20	0.28	n.d.	3.57	4.18	5.84	85

*Fruit bodies used for isolation of the mycelial strains used in this study

**Fig. 1:**
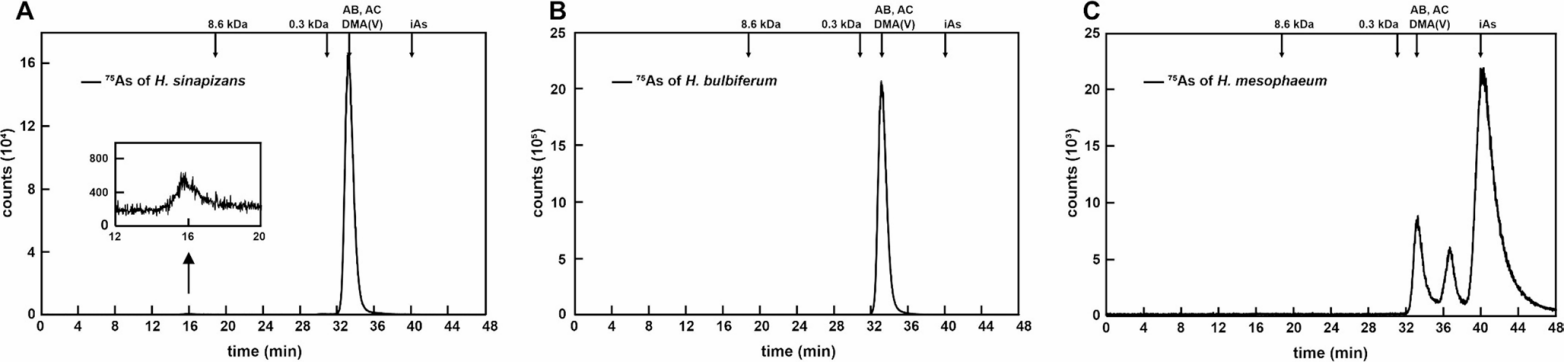
Size exclusion chromatography (SEC) fractionation of cell-free extracts from sporocarps of *Hebeloma sinapizans* (**A**), *Hebeloma bulbiferum* (**B**), and *Hebeloma mesophaeum* (**C**). Arrows indicate the elution maxima of the molecular mass standards or As species. The elemental content of individual fractions was measured using the AsNH_2_^+^ ion with ICP-MS. The chromatogram showing only the As species resolved on the column is shown in Supplementary Fig. 1

Size-exclusion chromatography (SEC) provides crucial insight into the molecular size distribution of As-containing biomolecules and helps determine if As is bound to any low-molecular-weight metabolites, peptides, or macromolecular complexes. Arsenic binding to peptide-type ligands (such as metallothioneins) has indeed been reported in other organisms [33–35], we have thus speculated that this phenomenon may also occur in the As-accumulating *Hebeloma* species. In all three sporocarp extracts, As was eluted into low-molecular-weight fractions (Fig. 1). This indicates that the As in these samples likely occurs as small-molecule As species, perhaps as free As species, rather than being bound to high-molecular-weight biomolecules. The similar elution profile across the different sporocarp extracts suggests a similar pattern of As handling among the studied mushrooms; however, in the extract of *H. sinapizans*, a small protein-associated As peak was detected, which may indicate a possible presence of a minor As-binding protein or peptide-type ligand (Fig. 1A).

To identify the As species present in the sporocarps, the mushroom tissue extracts were analysed using ion-pair reversed-phase inductively coupled chromatography with plasma mass spectrometer (IP-RP-ICP-MS). This analysis revealed pronounced differences in the As speciation of the fruit bodies. The As-accumulator *H. bulbiferum* predominantly contained DMA(V), accounting for over 99% of the extractable arsenicals (Table 1), with only trace amounts of TETRA, MA(V), and AC present. In contrast, *H. sinapizans* contained primarily AB (55%), followed by DMA(V) (28%) and TMAO (7%), along with trace amounts of MA(V), AC, and other unidentified arsenicals (Table 1). *H. mesophaeum* differed further, species being dominated by iAs (42%), followed by AC (32%) and DMA(V) (26%). These data exhibit the same pattern as those obtained from the SEC analysis, i.e., that most As is present as small-molecule As species rather than in higher-molecular-weight complexes. It can also be seen from Table 1 that As from *H. sinapizans* had lower extractability during the speciation analysis. This could be due to some of the As being associated with the protein fraction (Fig. 1).

The predominance of small-molecule As species observed in all analyzed extracts aligns with findings reported for other mushroom species. Previous studies have shown that As in fungi typically occurs in soluble, small-molecule forms, including both iAs and various organoarsenicals. Interspecific differences in As speciation patterns are also consistent with previous observations in other mushrooms [9, 36] and *Hebeloma* species [23]. These differences in speciation patterns suggest distinct metabolic pathways for As uptake, transformation, and sequestration that reflect species-specific biochemical adaptations rather than environmental variability.

### Arsenic Tolerance and Accumulation Assay of *Hebeloma* Mycelia

Before conducting experiments on mycelial cultures, the concentration range that the mycelia could tolerate had to be determined. Therefore, we conducted preliminary tolerance assays to evaluate the sensitivity of the three *Hebeloma* species to As(V). Although As(III) enters cells more efficiently, we chose to conduct our experiments using As(V) because it is the predominant form of As in most natural soils and aquatic environments [37, 38]. Thus, using As(V) better reflects the conditions that mycelia encounter in their natural habitats, providing a more environmentally relevant assessment of As tolerance and uptake.

**Fig. 2:**
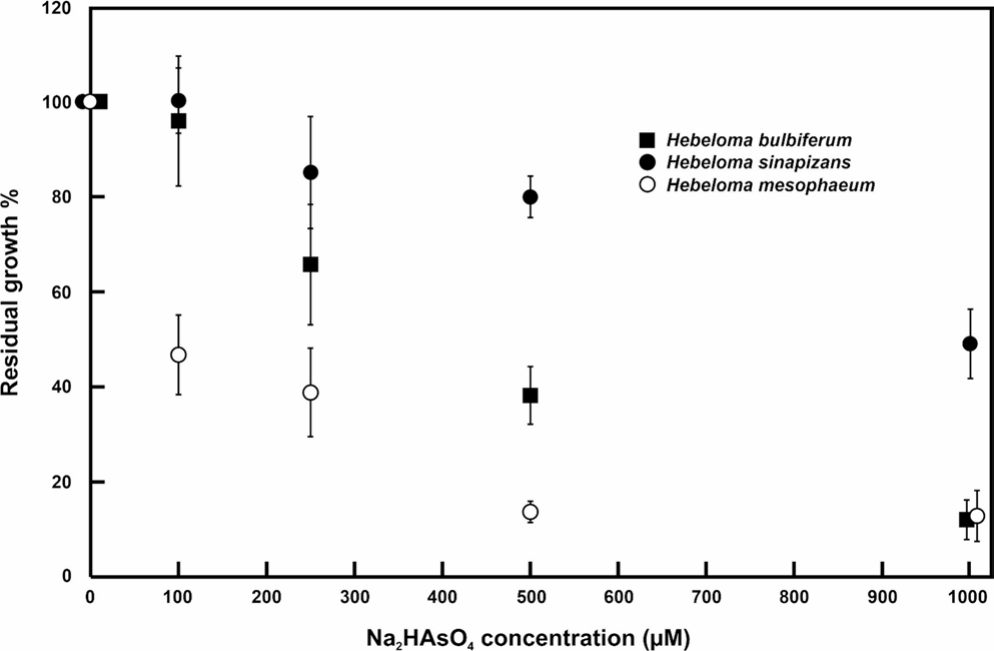
Arsenic tolerance assay of *Hebeloma* species mycelial isolates in the presence of various concentrations of Na_2_HAsO_4_ · 7H_2_O. Three replicate experiments were carried out at each concentration; the bars represent standard deviations

These experiments provided information for selecting suitable concentrations for subsequent tests. All three fungal species exhibited a clear, concentration-dependent inhibition of mycelial growth in response to As(V) (Fig. 2). *H. mesophaeum* was the most sensitive, showing substantial growth inhibition (biomass weight reduction to 14% compared to the 0 As(V) control) already at 500 µM Na_2_HAsO_4_ ·7H_2_O. In contrast, *H. bulbiferum* and *H. sinapizans* were more tolerant: at 500 µM Na_2_HAsO_4_ ·7H_2_O, *H. sinapizans* showed only slight growth reduction to 80%, while *H. bulbiferum* growth was reduced to 40%. At the highest tested concentration (1000 µM), *H. sinapizans* retained nearly 50% of its growth, while *H. bulbiferum* showed nearly complete inhibition (13%).

**Fig. 3:**
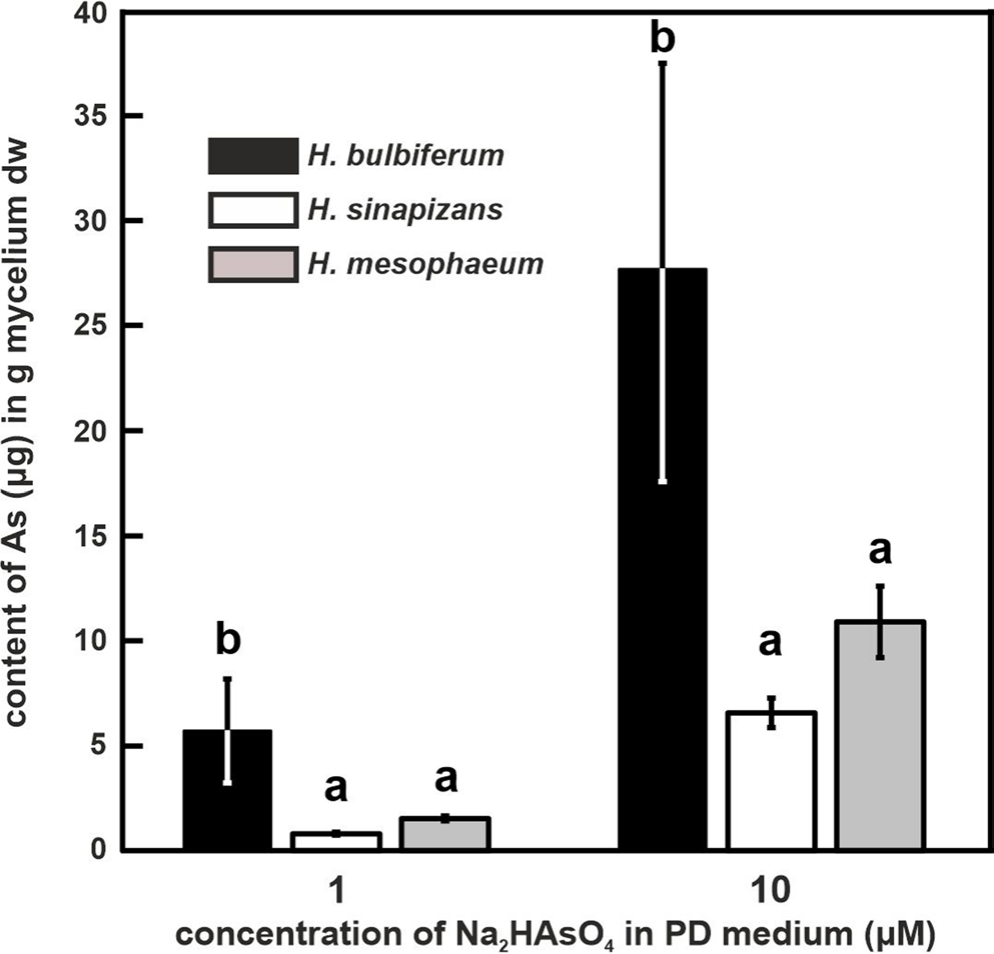
Accumulation capacity of *Hebeloma* mycelia. The plotted values represent the average of three biological replicates which were carried out at each Na_2_HAsO_4_ · 7H_2_O concentration, error bars represent standard deviation, and significant differences (*p* = 0.05, ANOVA followed by Tukey’s HSD test) in each treatment are indicated by different letters above the bars

Fungal arsenic (As) tolerance is hypothesized to be a multi-layered process beginning at the point of uptake, where, following established models like *Saccharomyces cerevisiae*, As(V) likely enters cells via high-affinity phosphate transporters such as Pho84p and Pho89p due to its structural homology with orthophosphate. This competitive uptake mechanism suggests that the eventual intracellular concentration is dictated by the efficiency of cellular transcription-translation mechanisms and the availability of competing nutrients [39]. Our findings reveal distinct, species-specific detoxification strategies within the *Hebeloma* genus. *Hebeloma bulbiferum*, an As accumulator, relies predominantly on methylation, with DMA(V) as its dominant intracellular species in fruit bodies. The moderate tolerance of this species suggests that while its uptake channels facilitate high accumulation, the internal machinery may become overwhelmed at higher concentrations *in vitro*. In contrast, *H. sinapizans* exhibits the highest tolerance despite being a not so strong accumulator both *in natura* (Table 1) and *in vitro* (Fig. 3), a robustness that may result from a highly regulated uptake system or specialized mechanisms like peptide binding, which is qualitatively supported by the high-molecular-weight peak observed during our SEC analysis (Fig. 1). Finally, *H. mesophaeum* is characterized by low accumulation and low tolerance, likely possessing inefficient uptake mechanisms and lacking complex sequestration strategies; instead, it appears to rely primarily on the simpler detoxification route of As(III) efflux via ACR3 transporters [5], which provides limited protection compared to the more sophisticated strategies of its congeners.

Based on the criteria that the levels must reflect realistic bioavailable As concentrations encountered under natural conditions and not inhibit fungal growth, we selected Na_2_HAsO_4_ ·7H_2_O concentrations of 1 µM, 10 µM, and 100 µM for the subsequent experiments.

### Arsenic Species Profiles and Accumulation Patterns in *Hebeloma* Mycelia

To elucidate As speciation profiles and accumulation patterns in *Hebeloma* mycelia, cultures of *H. bulbiferum*, *H. sinapizans*, and *H. mesophaeum* were grown on plates supplemented with As(V). After cultivation, the total As concentrations and As speciation were analyzed in the mycelium and the corresponding cultivation substrate (PD agar). Although biological replication per species was limited to two, clear fungal species-specific patterns appeared. Replicates within each *Hebeloma* species were more similar to each other than to replicates of the other *Hebeloma* species, especially in the terms of (i) total As uptake, (ii) As reduction/methylation proportion, and (iii) As distribution between agar and biomass. In the control (As supplemented, uninoculated, but cellophane covered) PD agar plates, total As was almost entirely detected as As(V), with a near 1:1 correlation between total As and As(V). No reduced or methylated species were detected. This confirms that As(V) remains chemically stable in the agar matrix for the whole cultivation period and that all observed transformation in fungal treatments is of biological origin rather than an abiotic artefact. On control plates (0 As added, inoculated), As in both mycelia and in agar under the mycelia was below the limit of detection (Supplementary Table 3).

**Fig. 4:**
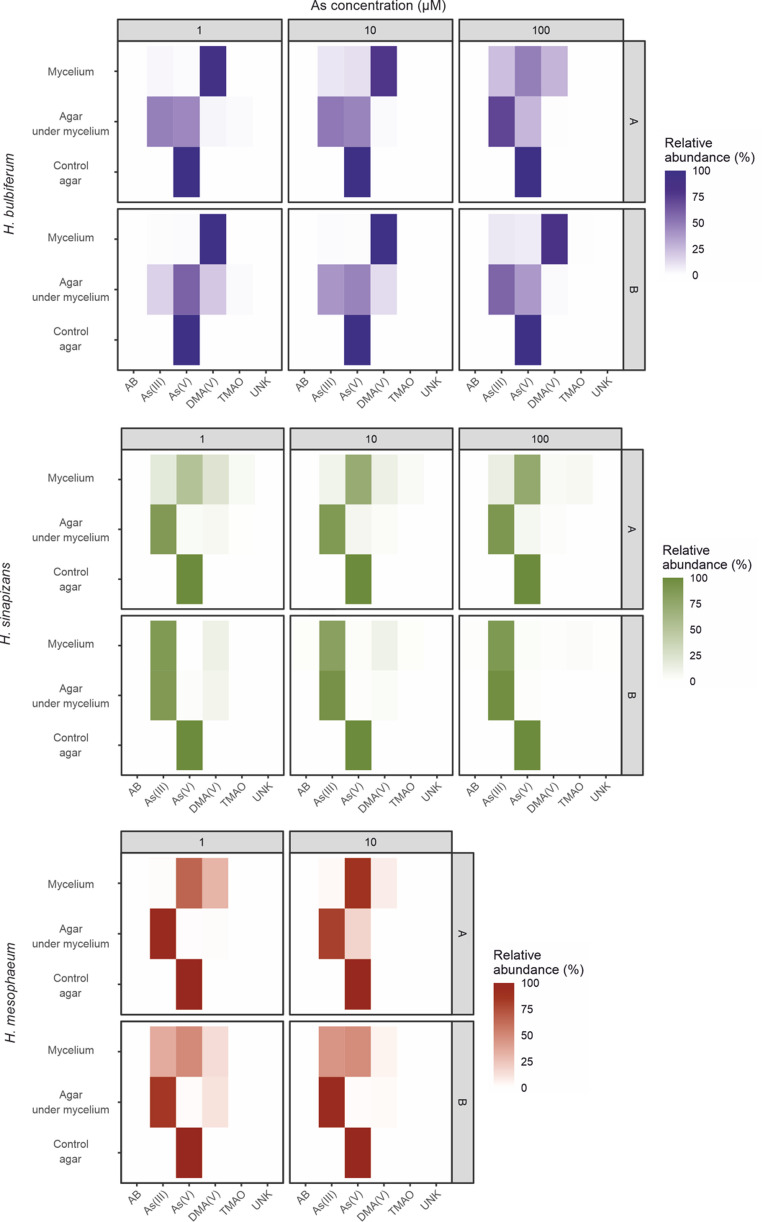
Comparative As speciation profiles across three *Hebeloma* species. The heatmap illustrates the relative abundance (%) of As species in fungal mycelia, the underlying PD agar, and uninoculated control PD agar across three exposure concentrations (1, 10, and 100 µM Na_2_HAsO_4_ · 7H_2_O). Data are presented for two biological replicates (A and B). Compound abbreviations: AB – arsenobetaine, As(III) – arsenite, As(V) – arsenate, DMA – dimethylarsenate, TMAO – trimethylarsine oxide, and UNK – unknown organoarsenical

Furthermore, all three *Hebeloma* species demonstrated the ability to transform iAs via two primary mechanisms: reducing As(V) to As(III), followed by its efflux from the cells, and converting iAs into various organic As compounds (Fig. 4; Supplementary Table 3). Notably, *H. sinapizans* and *H. mesophaeum* exhibited a higher degree of As(III) efflux than *H. bulbiferum*, indicating a more efficient As reduction and export. The ability of the fungi to reduce As(V) is not surprising because many organisms possess enzymatic systems dedicated to this process. For example, in bacteria, the *arsC* gene encodes an arsenate reductase [40, 41]. In yeast, *S. cerevisiae*, *arr2* serves a similar function [42]. In plants, the distinct *HAC1* gene mediates As(V) reduction [43]. Export of AsIII is also common and is supported by transport systems in diverse organisms. Transporters of the ACR3 family have been identified in plants, yeast, and bacteria. Several other transporter families contribute to this process in bacteria, with the ArsB playing a particularly prominent role [5, 44, 45]. However, these processes remain largely uncharacterized in macrofungi. Nevertheless, an ACR3-family transporter has recently been described in *H. sinapizans* and *H. bulbiferum*, where it is responsible for As export from the cells [46].

The mycelium of *H. mesophaeum* did not grow on the cellophane membrane in plates supplemented with 100 µM Na_2_HAsO_4_ · 7H_2_O, therefore, data are presented only for the lower concentrations. While *H. mesophaeum* and *H. sinapizans* primarily rely on As reduction and export (Fig. 4, Supplementary Table 3), *H. bulbiferum* mainly favors biotransformation into organoarsenic species, particularly DMA(V). Nevertheless, DMA(V) was the predominant organoarsenic species in all three investigated mushrooms. In some cases, *H. bulbiferum* exhibited particularly high proportions of DMA(V), accounting for over 90% of the total As content. Additionally, small amounts of TMAO were detected in *H. bulbiferum* and *H. sinapizans*. Furthermore, the mycelium of *H. sinapizans* contained trace amounts of AB and an unidentified compound. Thus, the speciation results obtained from the mycelia of all three species largely aligned with those observed in their corresponding fruit bodies (Table 1). The only compounds not detected in the mycelium were AC and TETRA in *H. bulbiferum* and *H. mesophaeum*, as well as MA(V) in *H. bulbiferum* and *H. sinapizans*. All of these organoarsenicals were present in the fruit bodies at low concentrations except for *H. mesophaeum*, where AC accounted for over 20% of total As.

### Fate of Organoarsenicals in Mycelia and Fruit Bodies

Since TMAO and DMA(V), but not AB, were detected in the agar, they can presumably be actively transported from the mycelia into the surrounding environment also in natural settings. Interestingly, a higher content of TMAO was detected in the *H. bulbiferum* cultivation medium than in the mycelium itself. The effective excretion of TMAO by the mycelium could possibly explain why TMAO was not detected in the fruit bodies of *H. bulbiferum*. In contrast, AB likely accumulates within the fungal cells, consequently becoming the predominant As species in the fruit bodies, as observed in many mushroom species [9 and references therein]. These findings are also supported by a previous study by Soeroes et al. [14], in which *A. bisporus* cultivated on wheat straw mixed with horse dung showed the presence of AB exclusively in the fruit bodies but not in the substrate, while iAs and DMA(V) were detected in both. The present experiment spanned only three weeks; however, the mycelium can persist for considerably longer periods in the natural environments, which may lead to the predominance of AB in the As composition of sporocarps. The compounds AC and TETRA can likely form only in the later stages of As detoxification, also because these two compounds constitute only a minor fraction of the As content in the fruit bodies. A high proportion of AC was observed only in sporocarps of *Sparassis crispa*, among the dozens of studied mushrooms, where it appeared to be one of the major As compounds [9 and references therein, 12].

The observed As speciation patterns suggest that the analysed *Hebeloma* species utilize a detoxification mechanism involving sequential reduction, methylation, and active transport. We propose that the initial transformation begins with the reduction of As(V) to As(III) within the cytosol, facilitated by As(V) reductases. This is particularly evident in *H. sinapizans* and *H. mesophaeum*, where the high concentrations of As(III) detected in the underlying agar suggest a primary reliance on active efflux via ACR3 transporters to remove toxic trivalent species before they disrupt intracellular processes. Indeed, the recent characterization of ACR3 transporters in these specific *Hebeloma* species provides a molecular basis for this “exclusion” strategy [46].

Beyond inorganic efflux, the presence of DMA(V) across all species indicates activation of the Challenger pathway. This mechanism involves the sequential oxidative methylation of As(III) into MMA(V) and subsequently DMA(V). The detection of TMAO in the mycelium of *H. bulbiferum* and *H. sinapizans* suggests a high-capacity methylation chain that extends to trimethylated forms. These transformations likely require a specialized enzymatic apparatus. In fungi, methyltransferases known as ArsM, originally characterized in bacteria, have been shown to produce DMA(V), as seen in the soil fungus *Westerdykella aurantiaca* [47]. Furthermore, because As methylation is a glutathione-dependent process, the known modulation of glutathione biosynthetic pathways by external As in the related species *Hebeloma cylindrosporum* [48] suggests that *Hebeloma* species possess a highly responsive metabolic framework for handling metalloid stress.

The detection of complex organoarsenicals, such as AB in *H. sinapizans* mycelia and AC in the surveyed fruit bodies, is particularly noteworthy. While the exact biosynthetic route for AB and AC in fungi remains under investigation, it is speculated to occur either via the degradation of arsenosugars or through a specialized pathway in which a dimethylated arsenic precursor is attached to a glycine- or choline-like molecule [11 and references therein]. However, we cannot disregard the potential role of the “mycosphere” or fungal endophytes in As transformations. Since AC was identified only in field-collected fruit bodies and not in axenic mycelial cultures, it is plausible that AC is accumulated from the environment, where it may be produced through external microbial activity or only during the fruiting body stage, as previously suggested for other macrofungi [49, 50]. This distinction between the metabolic potential of isolated mycelia and the chemical profile of field-collected fruit bodies highlights the complexity of As cycling in the symbiotic environment.

## Conclusions

The mycelia of *H. bulbiferum*,* H. sinapizans*, and *H. mesophaeum* are capable of reducing As(V) to the more toxic As(III), which is subsequently exported from the cells. This mechanism appears to represent the primary detoxification strategy employed by these mushrooms. Furthermore, the mycelia of the investigated *Hebeloma* species can transform iAs species into various organic forms. Remarkably, some of these organoarsenicals are efficiently exported into the surrounding environment. These findings suggest that the presence of organoarsenicals in soils and other terrestrial substrates may not be attributed solely to bacterial communities but also to fungal mycelia. We also demonstrated that arsenobetaine, typically produced by marine organisms, can be synthesized by some mushrooms. These results open new questions for future research, particularly regarding the identity of the enzymes responsible for As(V) reduction and As methylation. Understanding the specific molecular mechanisms and enzymes involved in these transformations, such as potential arsenate reductases or fungal ArsM methyltransferases, remains a key objective for elucidating the full detoxification pathway in these fungi.

## Supplementary Information

Below is the link to the electronic supplementary material.

Supplementary Material 1

## Data Availability

The datasets generated during and/or analyzed during the current study are available from the corresponding author on reasonable request.
