# Supplementary material for: Arsenic Speciation and Distribution in Three Hebeloma Species: Insights into Arsenic Handling and Transformation in Mycorrhizal Fungi
Source: Microb Ecol. 2026 May 6;89:134. doi: 10.1007/s00248-026-02780-9 (PMC13314834; doi:10.1007/s00248-026-02780-9)
Supplement: Supplementary file 1 [file 248_2026_2780_MOESM1_ESM.pdf]

Supplementary Figure 1: Size exclusion chromatography of the molecular mass standards. The element content of individual fractions was measured via $AsNH_2^+$ ion using ICP-MS.

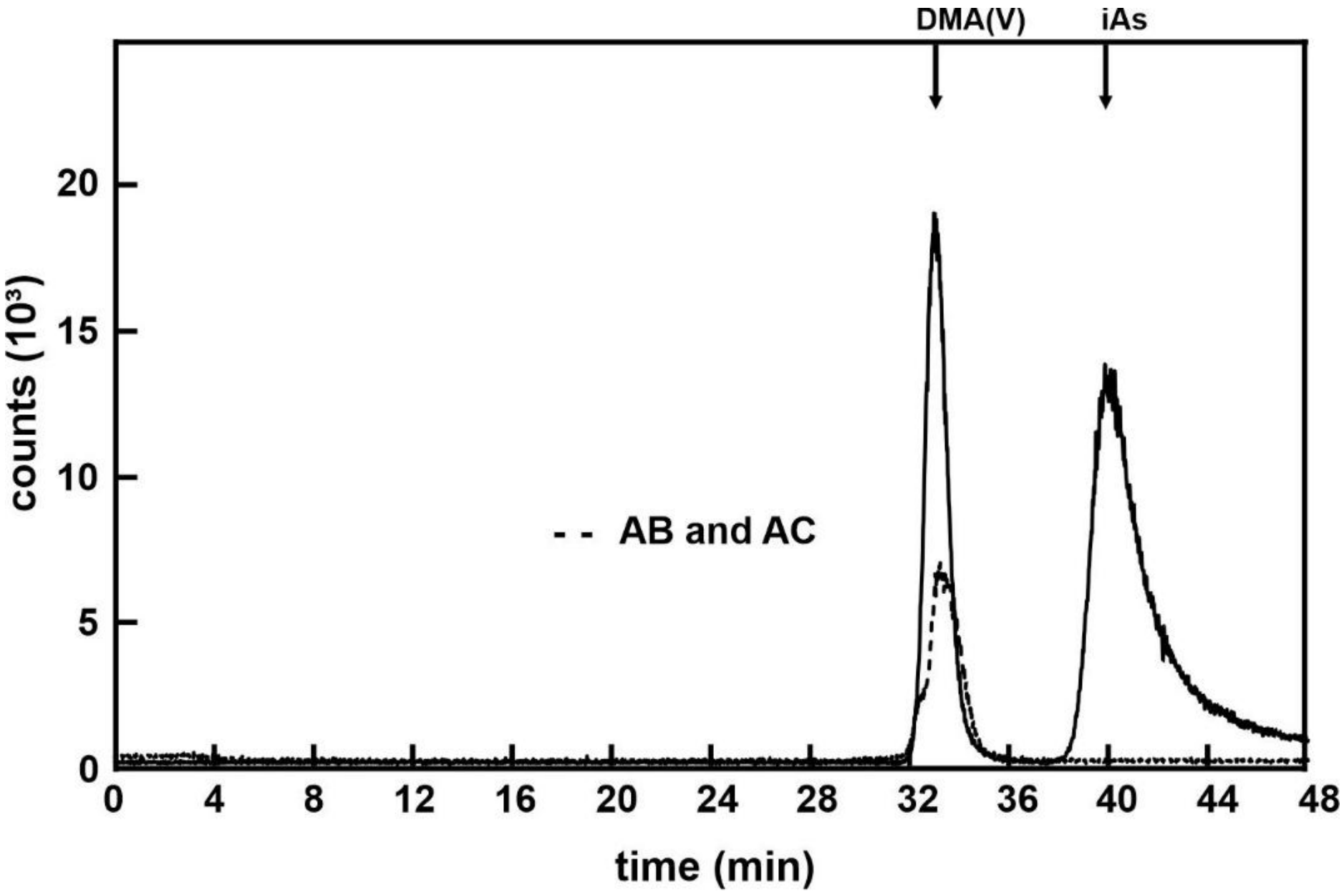

Supplementary Table 1. The total arsenic contents in fruit bodies of *Hebeloma bulbiferum* and *Hebeloma sinapizans* sampled at the investigated site (Prague-Velká Chuchle).

ID	coll. date	sample	As (mg kg^{-1} d. w.)
B 628	25 Sep 2010	*Hebeloma bulbiferum*	194
B 805a	15 Oct 2013	*Hebeloma bulbiferum*	343
B 805b	15 Oct 2013	*Hebeloma bulbiferum*	298
B 805c	15 Oct 2013	*Hebeloma bulbiferum*	434
B 805d	15 Oct 2013	*Hebeloma bulbiferum*	319
B 805e	15 Oct 2013	*Hebeloma bulbiferum*	275
B 1527	21 Oct 2016	*Hebeloma bulbiferum*	563
		**median**	**319**
B 629	25 Sep 2010	*Hebeloma sinapizans*	41.2
B 1525	21 Oct 2016	*Hebeloma sinapizans*	5.38
B 1526	21 Oct 2016	*Hebeloma sinapizans*	11.7
PAKO-101a	27 Oct 2020	*Hebeloma sinapizans*	12.2
PAKO-101b	27 Oct 2020	*Hebeloma sinapizans*	15.7
		**median**	**12.2**
-	-	soil 1	22.9
-	-	soil 2	21.8
-	-	soil 3	20.2
		**mean**	**21.6 ± 1.4**

Data for Standard Reference Materials (SRM)

**ID**	**Sample**	**Certified value (mg kg^{-1})**	**Measured As (mg kg^{-1})**
NIST 1566b	Oyster Tissue	7.65 ± 0.65	6.20
NIST 2711a	Montana II Soil	107 ± 5	103 ± 0.2

Supplementary Table 2. Arsenic content in individual fruit body parts of two *Hebeloma mesophaeum* collections from the investigated site (Řež near Prague).

ID	coll. date	*sample*	fruit body part	As (mg kg^{-1} d. w.)
B 269D	6 Nov 2007	*Hebeloma mesophaeum*	cap flesh	3.86
B 269L	6 Nov 2007	*Hebeloma mesophaeum*	lamellae	4.20
B 269T	6 Nov 2007	*Hebeloma mesophaeum*	stipe	1.10
B 346D	30 Oct 2008	*Hebeloma mesophaeum*	cap flesh	3.50
B 346L	30 Oct 2008	*Hebeloma mesophaeum*	lamellae	3.26
B 346T	30 Oct 2008	*Hebeloma mesophaeum*	stipe	0.55

Supplementary Table 3. Arsenic accumulation and speciation in *H. bulbiferum* (2A), *H. sinapizans* (2B), and *H. mesophaeum* (2C) mycelial isolates and agar medium. The experiment was set up in duplicate. All values are given in mg kg⁻¹ d. w.

**Table 2A**	sample	Final $Na_2HAsO_4 \cdot 7H_2O$) concentration in PD plate (µM)	Total As in sample (mg kg-1 d. w.)	Extraction efficiency (%)	As(III)	As(V)	DMA(V)	AB	TMAO	Unknown others
**Control**	PD agar	0	<	<	<	<	<	<	<	<
	PD agar	0	~	<	<	<	<	<	<	<
	PD agar	1	2.92	108	<	2.92	<	<	<	<
	PD agar	1	2.63	93	<	2.39	<	<	<	<
	PD agar	10	26.3	113	<	23.0	<	<	<	<
	PD agar	10	24.2	98	<	23.5	<	<	<	<
	PD agar	100	249	107	<	276	<	<	<	<
	PD agar	100	258	102	<	269	<	<	<	<
Hebeloma bulbiferum	mycelium	0	~	<	<	<	<	<	<	<
	mycelium	0	<	<	<	<	<	<	<	<
	mycelium	1	3.78	93	0.13	0.07	3.34	<	<	<
	mycelium	1	4.80	118	0.07	0.11	5.49	<	<	<
	mycelium	10	12.4	56	0.67	0.81	5.41	<	0.06	<
	mycelium	10	39.2	90	0.66	0.45	34.0	<	<	<
	mycelium	100	63.9	66	10.1	20.4	11.7	<	0.16	<
	mycelium	100	74.3	68	4.71	3.92	41.3	<	0.29	<
	PD agar under mycelium	0	<	<	<	<	<	<	<	<
	PD agar under mycelium	0	~	<	<	<	<	<	<	<
	PD agar under mycelium	1	3.73	122	2.18	2.05	0.19	<	0.11	<
	PD agar under mycelium	1	2.35	108	0.43	1.53	0.52	<	0.06	<
	PD agar under mycelium	10	32.9	68	11.4	10.4	0.50	<	0.10	<
	PD agar under mycelium	10	35.2	97	13.5	15.9	4.40	<	0.17	<
	PD agar under mycelium	100	340	75	184	68.9	1.75	<	0.49	<
	PD agar under mycelium	100	328	94	182	119	7.37	<	0.49	<

Table 2B

	sample	Final $Na_2HAsO_4 \cdot 7H_2O$ concentration in PD plate (μM)	Total As in sample (mg kg-1 d. w.)	Extraction efficiency (%)	As(III)	As(V)	DMA(V)	AB	TMAO	Unknown others
Hebeloma sinapizans	mycelium	0	~	<	<	<	<	<	<	<
	mycelium	0	<	<	<	<	<	<	<	<
	mycelium	1	0.81	81	0.12	0.34	0.15	<	0.04	<
	mycelium	1	0.87	47	0.35	<	0.05	<	<	<
	mycelium	10	6.50	37	0.24	1.72	0.32	<	0.12	<
	mycelium	10	7.31	43	2.52	0.09	0.36	0.07	0.07	<
	mycelium	100	86.9	36	4.42	23.4	1.57	<	2.15	<
	mycelium	100	101	44	39.5	1.53	0.89	0.88	1.38	0.34
	PD agar under mycelium	0	<	<	<	<	<	<	<	<
	PD agar under mycelium	0	~	<	<	<	<	<	<	<
	PD agar under mycelium	1	3.44	108	3.25	0.20	0.25	<	0.03	<
	PD agar under mycelium	1	3.44	97	2.92	0.08	0.32	<	<	<
	PD agar under mycelium	10	35.1	65	19.8	1.87	0.87	<	0.10	<
	PD agar under mycelium	10	34.0	103	32.7	0.49	1.56	<	0.17	<
	PD agar under mycelium	100	345	90	282	23.5	1.87	<	1.32	<
	PD agar under mycelium	100	315	100	310	4.29	0.86	<	1.33	<

Table 2C	sample	Final $Na_2HAsO_4 \cdot 7H_2O$ concentration in PD plate (µM)	Total As in sample (mg kg-1 d. w.)	Extraction efficiency (%)	As(III)	As(V)	DMA(V)	AB	TMAO	Unknown others
Hebeloma mesophaeum	mycelium	0	~	<	<	<	<	<	<	<
	mycelium	0	<	<	<	<	<	<	<	<
	mycelium	1	1.60	54	0.31	0.43	0.13	<	<	<
	mycelium	1	1.62	54	0.02	0.58	0.28	<	<	<
	mycelium	10	10.9	44	2.20	2.37	0.29	<	<	<
	mycelium	10	9.23	<	0.16	4.20	0.39	<	<	<
	PD agar under mycelium	0	<	<	<	<	<	<	<	<
	PD agar under mycelium	0	~	<	<	<	<	<	<	<
	PD agar under mycelium	1	3.45	80	2.36	0.06	0.35	<	<	<
	PD agar under mycelium	1	5.00	55	2.66	0.04	0.05	<	<	<
	PD agar under mycelium	10	40.6	61	23.4	0.54	0.74	<	<	<
	PD agar under mycelium	10	43.9	59	21.3	4.81	<	<	<	<
